# Examining a Thermodynamic Order Parameter of Protein Folding

**DOI:** 10.1038/s41598-018-25406-8

**Published:** 2018-05-08

**Authors:** Song-Ho Chong, Sihyun Ham

**Affiliations:** Department of Chemistry, Sookmyung Women’s University, Cheongpa-ro 47-gil 100, Yongsan-Ku, Seoul, 04310 Korea

## Abstract

Dimensionality reduction with a suitable choice of order parameters or reaction coordinates is commonly used for analyzing high-dimensional time-series data generated by atomistic biomolecular simulations. So far, geometric order parameters, such as the root mean square deviation, fraction of native amino acid contacts, and collective coordinates that best characterize rare or large conformational transitions, have been prevailing in protein folding studies. Here, we show that the solvent-averaged effective energy, which is a thermodynamic quantity but unambiguously defined for individual protein conformations, serves as a good order parameter of protein folding. This is illustrated through the application to the folding-unfolding simulation trajectory of villin headpiece subdomain. We rationalize the suitability of the effective energy as an order parameter by the funneledness of the underlying protein free energy landscape. We also demonstrate that an improved conformational space discretization is achieved by incorporating the effective energy. The most distinctive feature of this thermodynamic order parameter is that it works in pointing to near-native folded structures even when the knowledge of the native structure is lacking, and the use of the effective energy will also find applications in combination with methods of protein structure prediction.

## Introduction

Massive trajectory data are nowadays being generated routinely by atomistic biomolecular simulations owing to the development of the special-purpose computer, distributed computing networks, and clusters equipped with graphical-processing units^1–5^. This opened up the possibility to provide atomic-level details and insights behind many important biological processes that are difficult to address solely from experimental studies^6–10^. However, because of the high-dimensionality of the conformational space explored by biomolecules, a suitable transformation of the raw data is mandatory into a form that will make them easy to understand and interpret. Dimensionality reduction^11^, and subsequent clustering^12^ at a lower-dimensional subspace, are widely acknowledged methods that meet such a demand. Indeed, these methods constitute the first steps in constructing Markov state models that have received considerable attention in recent years^13–16^. Yet, the practical success of these approaches depends critically on the choice of good order parameters (or reaction coordinates; these terms will be used interchangeably) onto which the original high-dimensional time-series data are projected.

So far, geometric order parameters have been prevailing in dimensionality reduction and clustering of protein folding simulation trajectories. This is natural since those parameters are directly expressible by the coordinates generated by molecular dynamics simulations. Typical examples include the root mean square deviation (RMSD) to the native structure, the radius of gyration (*R*_*g*_), and the fraction (*Q*) of native amino acid contacts, whose use has been physically or empirically motivated. RMSD can be used also without knowing the native structure for the clustering purpose, which is done by computing pairwise RMSDs between simulated structures. More systematically derived collective reaction coordinates have also been commonly adopted, for example, those determined by the time-lagged independent component analysis (TICA)^17,18^ or the principal component analysis (PCA)^19^ of internal coordinates, which best characterize rare or large conformational fluctuations, respectively. These order parameters can be classified into two groups depending on whether external information other than raw simulation trajectories is utilized. For example, the knowledge of the native structure is necessary in computing the RMSD to that structure and *Q* values. On the other hand, no additional external information is required for computing *R*_*g*_, pairwise RMSDs, and the collective coordinates derived from TICA or PCA.

**Figure 1:**
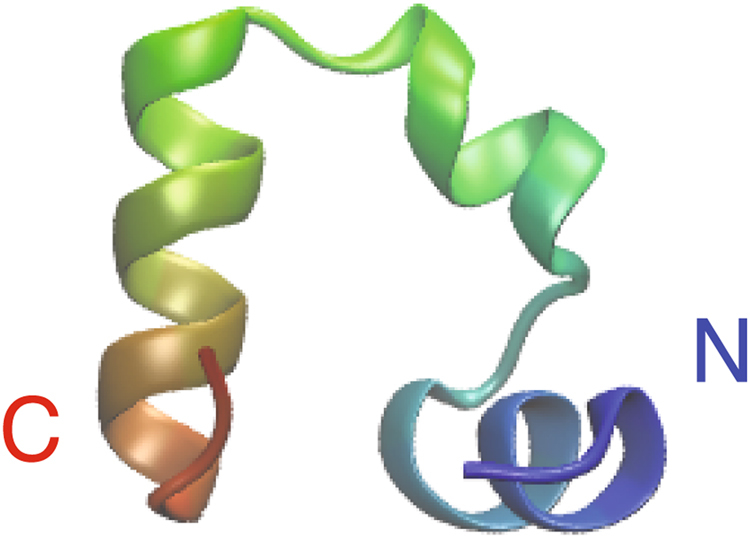
Native structure (PDB entry 1YRF) of the villin headpiece subdomain (HP35).

In this paper, we investigate the utility of a thermodynamic order parameter in protein folding studies. More specifically, we examine the suitability of the solvent-averaged effective energy^20,21^, to be denoted as *f* from here on, as a reaction coordinate of protein folding. This is a thermodynamic quantity since it involves the solvation free energy (i.e., averaging over solvent molecules), but like the geometric order parameters mentioned above, it can be defined and computed for individual protein conformations. To illustrate our main points, we shall deal with villin headpiece subdomain (HP35)^22^, one of the most popular systems for studying protein folding (see Fig. 1). Our work here is based on the ∼400 microsecond long folding-unfolding simulation trajectory of HP35 provided by the D. E. Shaw Research^23^. We first demonstrate that the effective energy *f* serves as a good order parameter of protein folding. We then rationalize this observation in terms of the funneledness of the underlying protein free energy landscape. We also show that an improved conformational space discretization is achieved by incorporating *f*. Finally, we showcase an analysis that clarifies the most distinctive feature of this thermodynamic order parameter, namely, that it can point to near-native structures even without the knowledge of the native structure. Thereby, we would like to establish the usefulness of this thermodynamic order parameter for investigating the protein folding.

## Results and Discussion

### Geometric vs. thermodynamic order parameters

**Figure 2:**
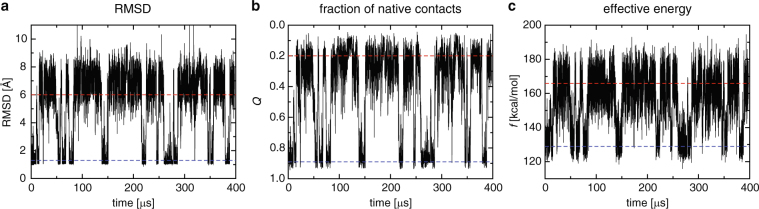
Order parameters of protein folding versus the simulation time. Cutoffs for defining the folded and unfolded states are shown by the dashed blue and red horizontal lines, respectively (see text for details). (**a**) C*α* RMSD to the native structure. (**b**) Fraction *Q* of native contacts, for which the ordinate is inverted so that the unfolded state is located at the top region and the folded state at the bottom region. (**c**) Effective energy *f*.

We start from overviewing the geometric order parameters commonly used in protein folding studies: C*α*-RMSD to the native (folded) structure (Fig. 2a) and the fraction (*Q*) of native amino acid contacts (Fig. 2b). We notice that the structural information of the native state is necessary in computing these parameters (see Methods). The folded state is characterized by small C*α*-RMSD and large *Q* values, whereas the unfolded state by large C*α*-RMSD and small *Q* values (the ordinate in Fig. 2b is inverted so that the unfolded state is located at the top region and the folded state at the bottom region as in Fig. 2a). Certain criteria are necessary for quantitative characterization of the folded state, unfolded state, and transition pathways between them. Cutoff values adopted in the previous studies for defining the folded and unfolded states of HP35 (1.3 Å and 6.0 Å for C*α*-RMSD^24^ and 0.89 and 0.20 for *Q*^25^) are shown by the dashed blue and red horizontal lines, respectively. The presence of a number of folding-unfolding transitions in the simulation trajectory is discernible from the time-variation of these parameters. A visual inspection of Fig. 2a,b indicates that C*α*-RMSD and *Q* are highly correlated. Indeed, the Pearson correlation coefficient of these two geometric order parameters takes a high value of *R* = 0.95 (Supplementary Fig. S1).

The solvent-averaged effective energy *f*–the thermodynamic order parameter that we argue in the present study–is introduced as follows^21,26^. Statistical properties of a protein (solute) dissolved in a solvent is determined by the partition function:

1 $${Z}_{{\rm{tot}}}=\int d{{\bf{r}}}_{u}\int d{{\bf{r}}}_{v}{e}^{-\beta [{E}_{u}({{\bf{r}}}_{u})+{E}_{uv}({{\bf{r}}}_{u},{{\bf{r}}}_{v})+{E}_{v}({{\bf{r}}}_{v})]}.$$

Here, ***r***_*u*_ and ***r***_*v*_ collectively denote the solute and solvent coordinates, respectively; *β* = 1/(*k*_B_*T*) is the inverse temperature; and *E*_*u*_, *E*_*uv*_, and *E*_*v*_ are respectively the intra-solute, solute-solvent, and solvent-solvent interaction energies. Since we are primarily interested in the protein configurations (**r**_*u*_) only, the solvent coordinates (**r**_*v*_) shall be averaged out. This can be done by introducing the solvation free energy

2 $${e}^{-\beta {G}_{u}^{{\rm{solv}}}({{\bf{r}}}_{u})}=\frac{1}{{Z}_{v}}\int d{{\bf{r}}}_{v}{e}^{-\beta [{E}_{uv}({{\bf{r}}}_{u},{{\bf{r}}}_{v})+{E}_{v}({{\bf{r}}}_{v})]}$$

in which ${Z}_{v}=\int d{{\bf{r}}}_{v}{e}^{-\beta {E}_{v}({{\bf{r}}}_{v})}$ is the partition function for the pure solvent. By combining equations (1) and (2), we obtain

3 $${Z}_{u}\equiv {Z}_{{\rm{tot}}}/{Z}_{v}=\int d{{\bf{r}}}_{u}{e}^{-\beta f({{\bf{r}}}_{u})}$$

Here enters the solvent-averaged effective energy, $f({{\bf{r}}}_{u})={E}_{u}({{\bf{r}}}_{u})+{G}_{u}^{{\rm{solv}}}({{\bf{r}}}_{u})$, that depends only on the protein configurations (***r***_*u*_). In fact, the quantity *f* is the genuine identity that defines the protein free energy landscape^20^. It is clear that the effective energy is unambiguously defined for each protein conformation. The gas-phase energy (*E*_*u*_) can be computed from the force field adopted in the simulation, where as a number of computational methods are available for the solvation free energy (${G}_{u}^{{\rm{solv}}}$). Here, we use the integral-equation theory for computing ${G}_{u}^{{\rm{solv}}}$ (see Methods and Supplementary Methods for details).

The time-variation of the effective energy *f* is shown in Fig. 2c. It is seen that, like C*α*-RMSD and *Q*, this thermodynamic variable also exhibits transitions between small and large values during the simulation. One understands from the physical ground (see also the next subsection) that the folded state is located at the small-*f* region, whereas the unfolded at the large-*f* region. Indeed, we obtain *f* = 128.9 and 165.9 kcal/mol, shown with the dashed blue and red horizontal lines in Fig. 2c, which were obtained as the average values for the protein conformations having *Q* = 0.89 and 0.20, respectively. We also find from scattered plots (Supplementary Fig. S1) that the effective energy *f* has a significant correlation both with C*α*-RMSD (*R* = 0.83) and with *Q* (*R* = 0.86). This indicates that *f* serves as a good reaction coordinate of folding.

### Funneled free energy landscape

**Figure 3:**
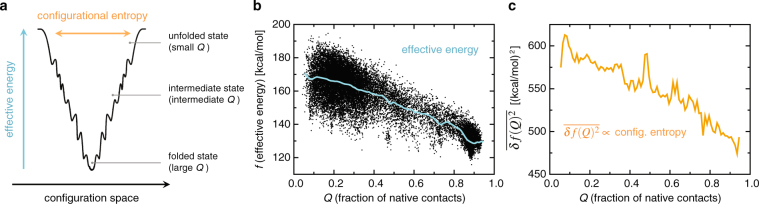
(**a**) Schematic illustration of the funneled free energy landscape. (**b**) Scatter plot of the effective energy (*f*) and the fraction (*Q*) of native contacts. The average effective energy $\overline{f(Q)}$ is drawn with the cyan solid curve. (**c**) Variance $\overline{\delta f{(Q)}^{2}}$ versus *Q*, which is proportional to the configurational entropy according to the energetic approach (see text for details).

Why does the effective energy (*f*) show such a significant correlation with the fraction (*Q*) of native contacts? In this regard, let us recall here the key assumption in a number of protein folding models, i.e., the “funneledness” of the free energy landscape^27–29^. This assumption states that the free energy landscape is globally funneled toward the native state, i.e., the effective energy *f* decreases as the native contacts are formed (see Fig. 3a). Computational results that support this assumption have been previously reported based on a simple model for the solvation free energy^30^, an analysis of the density of states for coarse-grained models^31^, and an explicit calculation of the solvation enthalpy^32^. Since we computed both the *Q* (Fig. 2b) and *f* (Fig. 2c) values along the folding-unfolding simulation trajectory, we can directly assess the funneledness of the folding landscape from a scatter plot of *f* and *Q*. This is shown in Fig. 3b, which demonstrates that the effective energy indeed tends to decrease as the native contacts are formed (i.e., as the folded state is approached). Thus, the funneled protein free energy landscape rationalizes the observed significant correlation between *f* and *Q*. Because of the significant correlation of *Q* and C*α*-RMSD mentioned above, this also explains that of *f* and C*α*-RMSD.

A digression might be in order here concerning the “width” of the landscape; in fact, the decrease in the width as the native state is approached must also occur to have a landscape that is funneled (see Fig. 3a). The width is quantified by the protein configurational entropy (*S*_conf_), that is, entropy associated with the protein’s internal degrees of freedom^27–29^. The decrease in the width can intuitively be understood as being due to the more ordered nature of the folded structures compared to the unfolded ones, but to the best of our knowledge, its demonstration based on microscopic approaches has not been done so far. To this end, we resort to the energetic approach^21,26,33^ according to which the configurational entropy for a given *Q* value can be expressed by the variance of *f*, $T{S}_{{\rm{conf}}}(Q)=(\beta \mathrm{/2)}\overline{\delta f{(Q)}^{2}}$ with $\delta f(Q)=f(Q)-\overline{f(Q)}$. Here, the bar denotes the average over the simulated protein conformations having a given value of *Q*. Since the variance $\overline{\delta f{(Q)}^{2}}$ measures the magnitude of the fluctuations in *f* along the vertical axis in the *f* vs. *Q* plot, the scatter plot shown in Fig. 3b indicates that the protein configurational entropy is indeed decreasing as the native contacts are formed. This is explicitly demonstrated in Fig. 3c. Thus, the effective energy *f* computed from the folding-unfolding simulation trajectory of HP35 exhibits the properties that are fully consistent with the funneled landscape paradigm.

### Conformational space discretization

**Figure 4:**
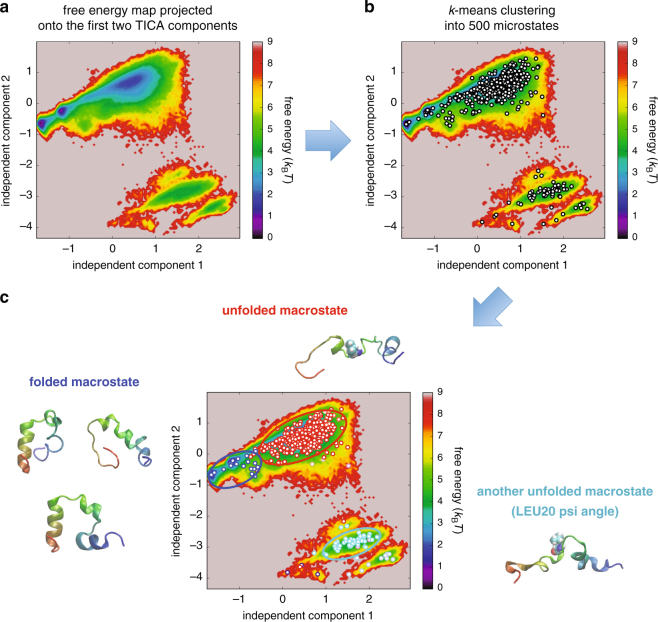
Protein conformational space discretization based solely on geometric order parameters. (**a**) Free energy map of the trajectory points projected onto the first two TICA components. (**b**) Locations of the 500 microstates (white circles) after applying the *k*-means clustering algorithm. (**c**) Ten macrostates identified by the PCCA algorithm distinguished by different colors. Three major macrostates discussed in the main text are enclosed by blue, red, and cyan ellipses. Representative sample structures of these macrostates are also displayed. LEU20 in the unfolded structures is shown with sphere representation.

Let us now investigate the effect of incorporating the thermodynamic order parameter in the protein conformational space discretization as an illustration of its use. This is done through constructing a Markov-state model (MSM)^14,34,35^ (see Methods for details). An MSM is defined with a set of discretized states and transition probabilities between them, and its construction requires the application of the clustering method to the low-dimensional subspace achieved via the dimensionality reduction. Before embarking on our main analysis, we first present the conventional conformational space discretization based solely on geometric order parameters. For this purpose, the folding-unfolding simulation trajectory of HP35 was first represented by the cosines and sines of the backbone *ϕ* and *ψ* angles, which were then projected onto a 10-dimensional subspace by applying the TICA algorithm^17,18^. A free energy map built from a histogram of the trajectory points projected onto the first two TICA components is shown in Fig. 4a. We next employed the *k*-means clustering to partition the trajectory points into 500 microstates, whose center positions are shown as white circles in Fig. 4b. Finally, after estimating the transition probabilities between the microstates using the simulation trajectory, the Perron cluster cluster analysis (PCCA) algorithm^36^ was adopted for coarse-graining the 500 microstates into 10 macrostates, which are distinguished by different colors in Fig. 4c.

We focus on the three major macrostates which are enclosed by blue, red, and cyan ellipses in Fig. 4c. Representative sample structures taken from these macrostates are also displayed. One infers from these structures that the blue macrostate is associated with the folded state, whereas the red and cyan macrostates with the unfolded state. We find that the structures in the latter two (red and cyan) unfolded macrostates differ mainly in the backbone *ψ* angle of LEU20 since these two macrostates are largely separated along the axis of the second independent component (Fig. 4c) and since this component is dominated by the contribution from the *ψ* angle of LEU20 (Supplementary Fig. S2). Concerning the folded (blue) macrostate, a close examination of the sample structures indicates that a number of unfolded structures are actually present in this macrostate. Indeed, the average C*α*-RMSD value to the native structure is found to be 2.6 Å for the blue macrostate, which is substantially larger than the cutoff (1.3 Å) for the folded state considered in Fig. 2a. Thus, a well-resolved folded macrostate could not be isolated based solely on the geometric order parameters.

**Figure 5:**
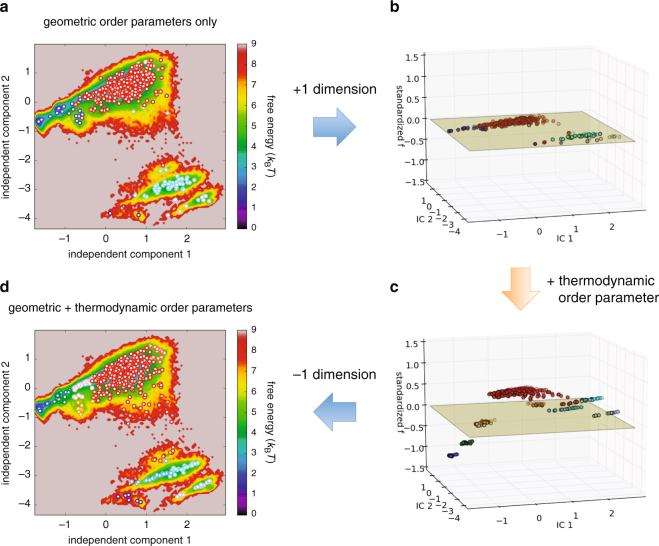
Procedures for incorporating the thermodynamic order parameter. (**a**) Protein conformational space discretization based solely on the geometric order parameters (see Fig. 4). (**b**) Addition of a thermodynamic dimension to prepare for incorporating the thermodynamic order parameter. (**c**) Incorporation of the thermodynamic order parameter in the standardized form $\hat{f}$ (see text for details). In this three-dimensional subspace spanned by the two TICA components (IC1 and IC2) and the thermodynamic coordinate $\hat{f}$, we carried out the *k*-means clustering into 500 microstates and then coarse grained them into 10 macrostates by applying the PCCA algorithm. These macrostates are distinguished by different colors. (**d**) The resulting protein conformational space discretization is projected back onto the original two-dimensional subspace.

Now we examine the effect of adding a thermodynamic dimension (*f*), expecting that its performance as a folding reaction coordinate contributes to gain a better conformational space discretization. Our procedures are illustrated in Fig. 5. We first took the first two independent components identified by the TICA algorithm. To these geometric coordinates, we added a thermodynamic coordinate, $\hat{f}$, which is obtained by standardizing the effective energy *f*, i.e., by a linear transformation such that it has zero mean and unit variance. (The independent components identified by the TICA algorithm also share this property as noted in Methods.) In this three-dimensional subspace spanned by the two TICA coordinates and the thermodynamic coordinate $(\,\hat{f})$, we carried out the *k*-means clustering to partition the trajectory points into 500 microstates. We then applied the PCCA algorithm to coarse-grain the 500 microstates into 10 macrostates. As a result, we find that the macrostates in the previous discretization (Fig. 5a) are more finely resolved in the new discretization (Fig. 5d); for example, whereas only two macrostates (colored blue and red) dominate the upper-left region in Fig. 5a, five macrostates (colored blue, green, orange, pink, and red) are discernible in the corresponding region of Fig. 5d (we recall here that, although it is represented in a two-dimensional map, the original discretization shown in Fig. 5a is done in a 10-dimensional subspace, and it is erroneous to consider that the improvement in Fig. 5d is achieved because of an increase in the subspace dimension).

**Figure 6:**
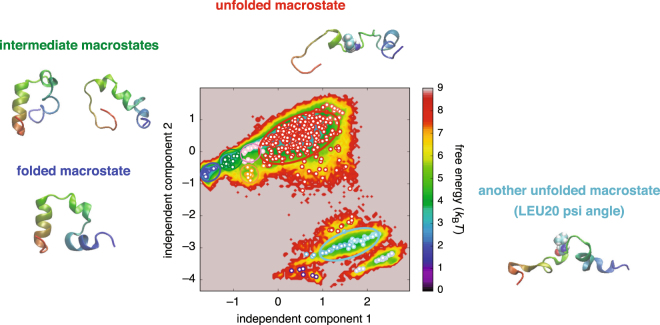
Protein conformational space discretization after incorporating the thermodynamic order parameter. Ten macrostates identified by the PCCA algorithm are distinguished by different colors. Major macrostates discussed in the main text are enclosed by blue, green, orange, pink, red and cyan ellipses, and some of their representative sample structures are also displayed. LEU20 in the unfolded structures is shown with sphere representation.

Representative macrostates after incorporating the thermodynamic order parameter are redrawn in Fig. 6 along with their sample structures. We find that the newly identified macrostates (colored green, orange, and pink) correspond to intermediate states between the folded (blue) and unfolded (red) macrostates. Indeed, the average C*α*-RMSD value to the native structure is 1.5 Å for the folded blue macrostate, which is comparable to the cutoff (1.3 Å) for the folded state used in Fig. 2a, whereas those values are 3.5, 5.1, and 5.1 Å for the intermediate green, orange, and pink macrostates, respectively, which are smaller than the cutoff (6.0 Å) for the unfolded state adopted in Fig. 2a. In a sense, this achievement of a better-resolved conformational space discretization is obvious since, in light of the significant correlation of *f* with C *α*-RMSD and *Q*, the effective energy *f* essentially carries the information on the “distance” to the native state. However, what is not obvious here is that this is achieved solely based on the raw simulation trajectory, i.e., without using the knowledge of the native structure.

### Distinctive feature of the thermodynamic order parameter

**Figure 7:**
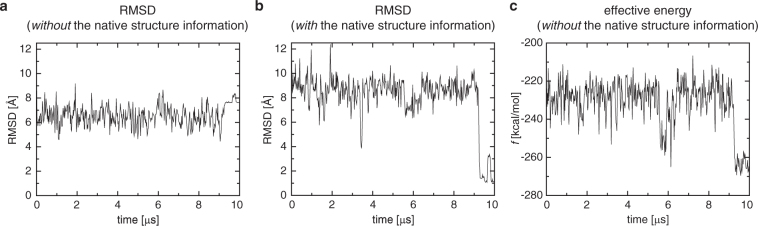
Order parameters of protein folding versus the simulation time computed for the WW domain (FiP35). (**a**) C*α* RMSD to an average structure. (**b**) C*α* RMSD to the native structure. (**c**) Effective energy *f*.

To further corroborate such a distinctive feature of *f*, we investigate here the first 10 microsecond portion of a folding simulation trajectory of the WW domain (FiP35), which is also provided by the D. E. Shaw Research^37^. The simulation was initiated from an unfolded structure and the folding occurs at ∼9 microsecond in this trajectory, but let us assume for a moment that we have no information on this nor on the native folded structure. Under this circumstance, one cannot compute *Q* values. In terms of C*α*-RMSD, what one can do would be to compute it with respect to an average structure. The result so obtained is displayed in Fig. 7a, from which one observes no clear-cut indication of a folding event. Of course, if we utilize the native structure information (PDB entry 2F21^38^), the presence of folding is evident as demonstrated in Fig. 7b, in which C*α*-RMSD to the native structure reaches ∼1 Å at ∼9 microsecond. The effective energy *f* computed along the trajectory, which is shown in Fig. 7c, also indicates a occurrence of folding at ∼9 microsecond, but remarkably this is accomplished even though no native structure information is used in computing *f*.

Free energy landscape concepts have been exploited not only in protein folding, but also in a variety of processes including biomolecular recognition, conformational changes upon ligand binding, and protein misfolding and aggregation^39–44^. Since the characteristics of the free energy landscape are naturally taken into account via the effective energy *f*, dimensionality reduction and clustering that incorporate *f* discussed in the present work will be useful also for investigating those interesting biomolecular processes. In particular, since the computation of *f* does not require any information on the native state, those approaches can equally be applied to analyzing intriguing dynamical processes involving intrinsically disordered proteins^45–47^ which do not have well-defined native structures.

## Conclusions

We investigate the utility of the solvent-averaged effective energy (*f*) as a possible order parameter in protein folding studies. This is a thermodynamic quantity since it is defined after averaging over solvent molecules, but like the root mean square deviation (RMSD) and the fraction (*Q*) of native contacts which have been commonly adopted as geometrical order parameters, it can be computed for individual protein conformations. We demonstrate that *f* serves as a good order parameter of protein folding, and this is rationalized by the funneledness of the protein free energy landscape. Remarkably, the thermodynamic order parameter *f* works better than the conventional geometrical order parameters in pointing to near-native structures when the knowledge of the native structure is lacking. The use of *f* will find applications not only for analyzing the protein conformational space, but also in combination with methods of protein structure prediction in ranking the predicted structures.

## Methods

### Computation of order parameters

We utilized the 397.5 microsecond long folding-unfolding simulation trajectory of HP35^23^ provided by the D. E. Shaw Research. Protein configurations saved with a 200 ps interval (i.e., 1,987,500 configurations in total) were subjected to the computation of the order parameters (C*α*-RMSD, *Q*, and *f*) discussed in the main text. The results shown in Fig. 2 are running averages over 20 ns (i.e., 100 configurations). C*α*-RMSD values were computed relative to the crystal structure of HP35 (PDB entry 1YRF^22^) excluding the first two and last two residues. The fraction (*Q*) of native amino acid contacts was calculated following ref.^25^. First, native contact pairs (*i*, *j*) are defined using the crystal structure. Here, a pair of non-hydrogen atoms *i* and *j* are considered to make a native contact if their distance is less than 4.5 Å and if the residues *θ*_*i*_ and *θ*_*j*_ they belong to satisfy |*θ*_*i*_−*θ*_*j*_| > 3. Then, *Q* can be computed from

4 $$Q({{\bf{r}}}_{u})=\frac{1}{N}\sum _{(i,j)}\frac{1}{1+\exp [\beta ({{r}}_{ij}({{\bf{r}}}_{u})-\lambda {r}_{ij}^{0})]}.$$

Here, *r*_*ij*_(**r**_*u*_) and ${r}_{ij}^{0}$ refer to the distances between *i* and *j* in a given protein conformation (**r**_*u*_) and in the crystal structure, respectively. We used the same values for the smoothing parameter *β* = 4 Å^−1^ and the factor *λ* = 1.8 as in ref.^25^. The computation of the effective energy for a given protein conformation **r**_*u*_, $f({{\bf{r}}}_{u})={E}_{u}({{\bf{r}}}_{u})+{G}_{u}^{{\rm{solv}}}({{\bf{r}}}_{u})$, proceeds as follows. We computed the gas-phase energy *E*_*u*_ from the force field adopted^23^. For the solvation free energy ${G}_{u}^{{\rm{solv}}}$, we employed the 3D-RISM theory^48^, whose details are provided in Supplementary Methods.

### Conformational space discretization

We employed PyEMMA 2^15^ for carrying out the steps in the MSM construction. The raw Cartesian coordinates in the simulation trajectory of HP35, taken with a 1 ns interval here, were first represented by the cosines and sines of the backbone *ϕ* and *ψ* angles. Next, the TICA algorithm^17,18^ was applied to those dihedral angles, with a 50 ns lag time and the output dimension of 10, to find a set of 10 slow coordinates (independent components) that define a lower-dimensional subspace. (Kinetic mapping scheme^49^ was not adopted here, and all the independent components have zero mean and unit variance.) Then, the clustering is carried out in the projected low-dimensional subspace. This is done using the *k*-means clustering, which was reported as one of the best algorithms in the MSM construction^50^, and we partitioned the trajectory points into 500 microstates. Then, the transition probabilities between these microstates were estimated using the simulation trajectory via the Maximum likelihood estimation with a 50 ns lag time. With the transition matrix, one can coarse-grain our system to get a simpler description. This is done using the PCCA algorithm^36^, and we coarse-grained the 500 microstates into 10 macrostates.

## Electronic supplementary material

Supplementary Information
