# Publisher Correction: Examining a Thermodynamic Order Parameter of Protein Folding

**DOI:** 10.1038/s41598-018-26490-6

**Published:** 2018-05-24

**Authors:** Song-Ho Chong, Sihyun Ham

**Affiliations:** Department of Chemistry, Sookmyung Women’s University, Cheongpa-ro 47-gil 100, Yongsan-Ku, Seoul, 04310 Korea

Correction to: *Scientific Reports* 10.1038/s41598-018-25406-8, published online 08 May 2018

The original version of this Article was published with an incorrect version of the Supplementary Information file due to a technical error. This has now been corrected in the HTML; the PDF version of the paper was correct from the time of publication.

