# Supplementary material for: Examining a Thermodynamic Order Parameter of Protein Folding
Source: Sci Rep. 2018 May 8;8:7148. doi: 10.1038/s41598-018-25406-8 (PMC5940758; doi:10.1038/s41598-018-25406-8)
Supplement: Supplementary file 1 — Supplementary Information [file 41598_2018_25406_MOESM1_ESM.pdf]

Supplementary Information for

Examining a Thermodynamic Order Parameter of Protein Folding

Song-Ho Chong and Sihyun Ham*

Department of Chemistry, Sookmyung Women's University,

Cheongpa-ro 47-gil 100, Yongsan-Ku, Seoul 04310, Korea

Corresponding author:

*Sihyun Ham

Address Department of Chemistry, Sookmyung Women's University

Cheongpa-ro 47-gil 100, Yongsan-Ku, Seoul 04310, Korea

Email sihyun@sookmyung.ac.kr

Phone +82-2-710-9410

Fax +82-2-2077-7321

Calculation method of solvation free energy

To each of the simulated solute configurations, we applied the three-dimensional reference interaction site model (3D-RISM) theory[S1] to compute the solvation free energy G_u^{solv}. The 3D-RISM theory is an integral-equation theory for obtaining the 3D distribution function $g_\gamma(\mathbf{r})$ of the water site γ at position $\mathbf{r}$ around the solute. In this theory, the distribution function is obtained by self-consistently solving the 3D-RISM equation

$$h_\gamma(\mathbf{r}) = \sum_{\gamma'} \int d\mathbf{r}'\, \chi_{\gamma\gamma'}(|\mathbf{r}-\mathbf{r}'|)\, c_{\gamma'}(\mathbf{r}') \tag{S1}$$

and the approximate closure relation

$$h_\gamma(\mathbf{r}) = \begin{cases} \exp[d_\gamma(\mathbf{r})] - 1 & \text{for} \quad d_\gamma(\mathbf{r}) \le 0 \\ d_\gamma(\mathbf{r}) & \text{for} \quad d_\gamma(\mathbf{r}) > 0 \end{cases} \tag{S2}$$

in which $d_\gamma(\mathbf{r}) = -u_\gamma(\mathbf{r})/(k_{\mathrm{B}}T) + h_\gamma(\mathbf{r}) - c_\gamma(\mathbf{r})$. Here $h_\gamma(\mathbf{r}) = g_\gamma(\mathbf{r}) - 1$ and $c_\gamma(\mathbf{r})$ are the total and direct correlation functions, respectively; $\chi_{\gamma\gamma'}(r)$ denotes the site-site solvent susceptibility function which can be obtained either from simulations or integral-equation calculations; and $u_\gamma(\mathbf{r})$ is the solute-solvent interaction potential for a given solute configuration. We used the same numerical procedure as described in ref S1 to solve the above equations. Solvation free energy can then be computed from the following analytical expression:

$$G_u^{\mathrm{solv}} = \rho k_{\mathrm{B}}T \sum_\gamma \int d\mathbf{r}\, \left[\frac{1}{2}h_\gamma(\mathbf{r})^2\Theta(-h_\gamma(\mathbf{r})) - c_\gamma(\mathbf{r}) - \frac{1}{2}h_\gamma(\mathbf{r})c_\gamma(\mathbf{r})\right] \tag{S3}$$

Here, ρ is the average solvent number density, and Θ is the Heaviside step function.

References

(S1) Imai, T.; Harano, Y.; Kinoshita, M.; Kovalenko, A.; Hirata, F. A Theoretical Analysis on Hydration Thermodynamics of Proteins. *J. Chem. Phys.* **2006**, *125*, 024911.

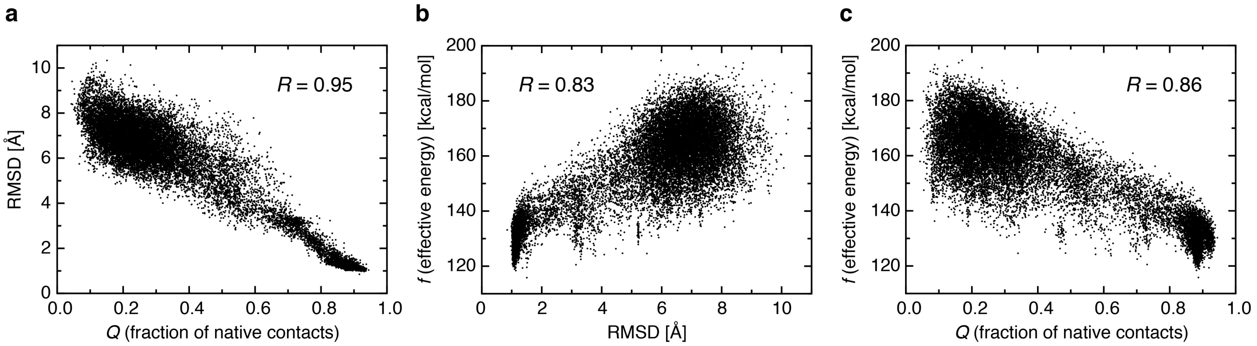

Figure S1: Scatter plots of (a) Cα-RMSD to the native structure versus the fraction of native amino acid contacts Q, (b) effective energy f versus Cα-RMSD, and (c) f versus Q. Pearson correlation coefficient is also displayed in each panel.

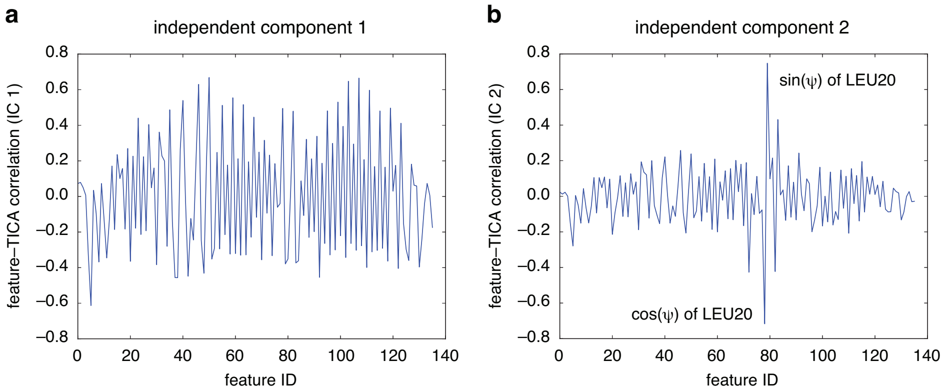

Figure S2: Correlation between the TICA component and features (cosines and sines of backbone ϕ and ψ angles) for the first (a) and second (b) TICA components.
